# Oral Administration of Nicotinamide Mononucleotide Increases Nicotinamide Adenine Dinucleotide Level in an Animal Brain

**DOI:** 10.3390/nu14020300

**Published:** 2022-01-12

**Authors:** Chidambaram Ramanathan, Thomas Lackie, Drake H. Williams, Paul S. Simone, Yufeng Zhang, Richard J. Bloomer

**Affiliations:** 1 College of Health Sciences, University of Memphis, Memphis, TN 38152, USA; talackie@memphis.edu (T.L.); yzhang24@memphis.edu (Y.Z.); rbloomer@memphis.edu (R.J.B.); 2 Department of Chemistry, University of Memphis, Memphis, TN 38152, USA; dhwllms3@memphis.edu (D.H.W.); psimone@memphis.edu (P.S.S.)

**Keywords:** NAD^+^, NMN, brain, oral gavage, mice

## Abstract

As a redox-sensitive coenzyme, nicotinamide adenine dinucleotide (NAD^+^) plays a central role in cellular energy metabolism and homeostasis. Low NAD^+^ levels are linked to multiple disease states, including age-related diseases, such as metabolic and neurodegenerative diseases. Consequently, restoring/increasing NAD^+^ levels in vivo has emerged as an important intervention targeting age-related neurodegenerative diseases. One of the widely studied approaches to increase NAD^+^ levels in vivo is accomplished by using NAD^+^ precursors, such as nicotinamide mononucleotide (NMN). Oral administration of NMN has been shown to successfully increase NAD^+^ levels in a variety of tissues; however, it remains unclear whether NMN can cross the blood–brain barrier to increase brain NAD^+^ levels. This study evaluated the effects of oral NMN administration on NAD^+^ levels in C57/B6J mice brain tissues. Our results demonstrate that oral gavage of 400 mg/kg NMN successfully increases brain NAD^+^ levels in mice after 45 min. These findings provide evidence that NMN may be used as an intervention to increase NAD^+^ levels in the brain.

## 1. Introduction

The oxidized form of nicotinamide adenine dinucleotide (NAD^+^) is an electron carrier and signaling molecule found virtually in every cell in our body and is involved in a broad spectrum of biological processes [1,2]. NAD^+^ is a coenzyme for redox reactions, functioning as a critical regulator to maintain physiological processes [3]. Changes in NAD^+^ levels are reported during aging and age-related diseases, such as diabetes, cancer, neurodegeneration, and cardiovascular diseases [4]. Preclinical studies suggest that NAD metabolism and/or NAD^+^/NADH redox balance are potential therapeutic targets [5,6].

Numerous approaches have been employed to manipulate NAD^+^ levels in cells and in vivo [7]. NAD^+^ levels can be increased by activating enzymes that stimulate the synthesis of NAD^+^ [8]. It can also be done by inhibiting an enzyme (CD38) that degrades NAD^+^ [9,10] and also by supplementing with NAD precursors, including nicotinamide riboside (NR) and nicotinamide mononucleotide (NMN) [6]. Although nicotinamide/nicotinic acid and nicotinamide ribose (NR) can both be used as precursors for synthesizing NAD^+^, NMN is a predominant precursor in mammals [11]. NMN is a bioactive nucleotide containing a nicotinamide, ribose, and a phosphate group [12]. Naturally, the NMN is found in small amounts in fruits and vegetables, such as avocados, broccoli, cabbage, edamame, and cucumbers. Taken orally, NMN can be rapidly absorbed and converted to NAD^+^ by the enzyme nicotinamide/nicotinic acid mononucleotide adenylyltransferase (NMNAT) [6]. In several studies, supplementation with NMN suppressed age-related adipose tissue inflammation, enhanced insulin secretion and insulin action, improved mitochondrial function, and improved neuronal function in the brain [6,13,14].

Studies from rodents have demonstrated that physiological NMN administration effectively enhances NAD^+^ biosynthesis in multiple peripheral tissues, including the pancreas [11], liver [11,15,16], adipose tissue [17], heart [18,19], skeletal muscle [10], kidney [20], eyes [21], and blood vessels [22]. Even though it is not known how NMN can cross the blood–brain barrier (BBB) [6], intraperitoneal NMN administration rapidly increases NAD^+^ levels in the hippocampus and hypothalamus brain regions [13,23], indicating the NMN could pass through the BBB and act as a substrate for NAD^+^ biosynthesis in the brain regions. However, direct evidence of manipulation of NAD^+^ levels in the brain through oral NMN administration is still lacking. Consequently, in the present study, we administered NMN through oral gavage and tested whether it changed NAD^+^ levels in the brain tissue in mice. We hypothesized that oral administration of NMN would rapidly increase NAD^+^ levels in the brain. This study would help us to understand the effects of oral NMN administration on brain NAD^+^ levels, which would allow us to evaluate the possibility of using oral NMN administration to increase NAD^+^ levels in vivo.

## 2. Materials and Methods

### Animals

This study was conducted under the University of Memphis IACUC permit (#0872). Twelve, four-week-old male C57BL/6J mice were purchased from the Jackson Laboratory. Our sole use of healthy, male mice should be viewed as a limitation of this work. Mice were housed in the animal facility with 12 h light/12 h dark cycles with ad libitum food and water for a one week acclimation period. Mice were then separated into a control group and the NMN group. NMN administration was accomplished by using CalerieHealth^TM^ SOD+, a 100% β-NMN isoform supplement (CalerieHealth™, Anaheim, CA, USA). Tablets of CalerieHealth^TM^ SOD+NMN were dissolved in PBS at a concentration of 720 mg/mL of NMN. NMN group mice (*n* = 6) received NMN (400 mg/kg) through oral gavage. For example, for an average 4-week-old mouse weighed at 16 g, 88.9 μL of CalerieHealth^TM^ SOD+NMN solution was administrated through oral gavage. The control group mice (*n* = 6) received the same volume of PBS compared to their NMN group counterparts. After 45 min, mice were euthanized by CO_2_, and the whole brain was quickly dissected and snap-frozen in liquid nitrogen. Skeletal muscles (leg muscles) were dissected and snap-frozen in liquid nitrogen. Tissues were stored at −80 °C for future analysis of NAD^+^ (in brain, as described below) and SOD (skeletal muscle, via Western blot). This latter analysis was not a primary purpose of the study, and no significant differences were detected due to the small sample size; hence, data are not shown.

NAD^+^ in brain tissues was extracted through acid extraction according to Yoshino and Imai [24]. Briefly, pre-chilled 10% HClO_4_ solution was added in the left side of each brain at a 1:10 ratio (tissue weight: HClO_4_ volume) and homogenized using a Polytron Homogenizer on ice. Then, the homogenates were centrifuged at maximal speed (~15,000× *g*) for 5 min at 4 °C. Supernatants were transferred to new tubes, and one-third volume of 3 M K_2_CO_3_ was added and mixed well. Samples were centrifuged again at maximal speed (~15,000× *g*) for 5 min at 4 °C. Supernatants were transferred to new tubes and frozen at −80 °C until HPLC-Mass Spectrometry measurements.

NAD^+^ levels in samples were measured using HPLC-Mass Spectrometry according to Mills, et al. [15]. For the separation and analysis, a Waters AQUITY UPLC with a Waters Quattro Micro Triple-Quadrupole Mass Spectrometer was operated in binary gradient mode. The binary gradient employed was comprised of 5.0 mM ammonium formate in an aqueous solution for solvent A and methanol for solvent B. The initial conditions were 100% solvent A and held for 1 min. From 1 min to 3 min, the concentration of solvent A decreased to 30% of initial with the remaining 70% as solvent B. This was held for 1 min. From 4 min to 5 min, solvent composition returned to initial conditions and held until the end of the run. The mass spectrometer was operated in ESI positive mode with multiple reaction monitoring (MRM) using the 664 *m*/*z* > 428 *m*/*z* transition for NAD^+^. The acquisition window for NAD^+^ was 2.0–3.5 min. The operating voltages were as follows: the capillary voltage was 0.8 kV, the cone voltage was 30 V, and the collision cell voltage was 30 V. A stock standard solution of NAD+ was prepared at a concentration of 0.2 mg/mL and serially diluted to produce seven calibration standards in the range of 100 to 10,000 µg/L. A check standard was analyzed seven consecutive times at 500 µg/L to determine the method detection limit [25], accuracy and precision [26]. The method detection limit was determined by multiplying the standard deviation of the reported check standard conversation by a Student’s t-value at the 98% confidence level for n − 1 degrees of freedom. Accuracy was estimated as mean % recovery of the reported check standard concentration. Precision was estimated as % relative standard deviation of the reported check standard concentration. The method detection limit for NAD^+^ was 77.4 µg/L, the accuracy was estimated as 117%, and the precision was estimated as 4.2% RSD.

Results are expressed as mean ± SEM, and statistical differences were calculated using a paired *t*-test with IBM SPSS.

## 3. Results

**Figure 1:**
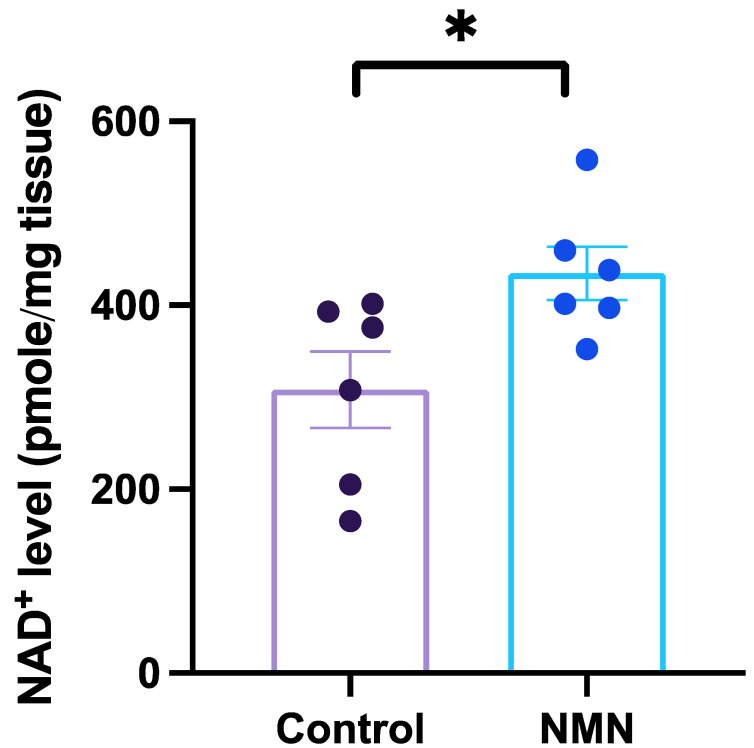
Oral gavage administration of nicotinamide mononucleotide (NMN) increases nicotinamide adenine dinucleotide (NAD^+)^ in brain tissue of mice. Data are expressed as mean ± SEM. The histogram depicts the NAD^+^ level in the brain tissues of NMN (400 mg/kg) and PBS (control) administrated mice 45 min after oral gavage. * indicates *p* = 0.0347.

For this study, we have documented an increase of NAD^+^ levels in brain tissues following oral administration of NMN (Figure 1). The dosage of NMN was 400 mg/kg, within the range of dosages (300–500 mg/kg) used in previous studies [6]. The mice treated with NMN significantly increased the NAD^+^ levels more than 40% compared to their control counterparts (*t*_5_ = 2.878, *p* = 0.0347; Figure 1). The results from the present study demonstrate that NMN can increase brain NAD^+^ levels rapidly, likely via passing through the blood–brain barrier (Note: since we did not measure blood NMN levels, we made the assumption that oral administration first led to increased blood NMN and then subsequently yielded the increase in brain NAD^+^.).

## 4. Discussion

Considering our data, similar results have been observed in mice that received NMN (300 mg/kg) and brain tissues collected 60 min after oral gavage [15]. Similar to this study, Mills et al. [15] had observed a slight increase in NAD^+^ levels, though not significantly, in the cortex of mice compared with their controls. These results indicate that the higher dosage (400 mg/kg) employed in the present study would be more beneficial to manipulate brain NAD^+^ levels. Moreover, the dosages and treatment time might also be different depending on the tissues of interest and administration methods. For example, oral administration of NMN (300 mg/kg) in mice has been shown to increase NAD^+^ levels in plasma as quickly as 2.5 min and return back to the original levels at 15 min. However, liver and skeletal muscle changes were not observed until 15 min [15]. On the other hand, administrating NMN (500 mg/kg) intraperitoneally increased NAD^+^ levels in the liver, pancreas, and white adipose tissue in 15 min [11]. Hence, dosages and treatment time of NMN targeting an increase in NAD^+^ levels should be optimized for administration methods and tissues of interest. As cellular NAD^+^ homeostasis is regulated by the balance between NAD^+^ consuming enzymes and the NAD^+^ synthesizing enzymes, NMN effects are only short-lived [3,27]. It is well known that NMN is soluble in water and saline and is taken up more efficiently through the plasma membrane. The cells immediately convert the NMN into NAD^+^. The NAD^+^ consuming enzymes, such as sirtuins, poly-ADP-ribose polymerases (PARPs), and CD38/157 ectoenzymes, used the excess NAD^+^ for many biological processes and reduced the extra level of NAD^+^ to the average physiological level [28,29]. At the same time, the biosynthetic pathway also plays a primary role in maintaining the physiological level of NAD^+^. Among three NAD^+^ biosynthetic pathways, the salvage pathway is the most predominant, and it is controlled by an endogenous circadian clock. The cellular circadian clock senses the level of NAD^+^ and activates the NAMPT, a rate-limiting enzyme for NAD^+^ synthesis, and controls the salvage pathway by generating a homeostatic level of the NAD^+^ at the cellular level [30,31].

The result suggests that NMN may offer a broad application and therapeutical potential. A growing body of evidence shows that NMN has beneficial effects on various neurodegenerative mice models, such as Alzheimer’s disease, Parkinson’s disease, and cognitive deficit models (Table 1). Age-related decline of cellular NAD^+^ levels contributes to various age-related diseases, especially neurodegenerative diseases, including Alzheimer’s, Parkinson’s, and Retinal degenerative diseases [1]. NAD^+^ sirtuin axis plays an important role in preventing neuronal cell death, which is commonly observed in these neurodegenerative disorders. In mice, cortex and hippocampal NAD^+^ levels decrease in the early age of Alzheimer’s and Parkinson’s disease [32,33]. In Alzheimer’s disease, the decrease of NAD^+^ levels has been shown to be associated with decreased activity of nicotinamide phosphoribosyltransferase (NAMPT) [34]. NAMPT is a vital enzyme in cells that convert nicotinamide to NMN, where NMN can be further converted to NAD^+^ by nicotinamide/nicotinic acid mononucleotide adenylyltransferase [35].

Consequently, NMN treatment would bypass the decreased NAMPT activity in Alzheimer’s disease patients, which can be used as a potential treatment. Studies with Alzheimer’s disease mouse models have documented that NMN supplementation reduces neural death and enhances cognitive function [14,38,40]. Similarly, in retinal degenerative diseases, retinal degeneration and blindness were often caused by malfunction of retina-specific NAMPT [21]. In this study, the administration of NMN could be used to restore retinal function and rescue vision. Moreover, NMN had also shown significant beneficial effects by attenuating neuronal cell apoptosis and improving energy metabolism in a cellular model of Parkinson’s disease. Thus, NAD^+^ metabolism is recognized as an attractive target for nutritional intervention against various neuronal disorders. NAD^+^ precursors, such as NMN, could be used as a potent supplement against various age-related neurodegenerative diseases.

Besides the brain, experimental evidence supports the use of short-term administration of NMN for therapeutic effects on metabolic diseases, cardiovascular complications, and mitochondrial dysfunctions [27]. For example, NMN improves impairments in glucose-stimulated insulin secretion in both genetic mouse models and aged wild-type mice [44]. NMN supplementation reduced adiposity in mice, and it had stronger effects on liver fat catabolism and synthesis even in comparison to exercise [45]. The NMN-mediated increase of NAD^+^ levels has been shown to protect the heart from ischemia/reperfusion injury, sustains the neural stem/progenitor cell population, reestablishes skeletal muscle mitochondrial function and arterial function in aged mice, and facilitates mitochondrial function [46,47]. These results indicate that NMN can be quickly absorbed, efficiently transported in blood circulation, and taken up and converted to NAD^+^ in different tissues. Enhancing NAD^+^ biosynthesis with NMN may be an efficient therapeutic intervention against many disease conditions.

As a result of the potential high efficacy and benefits of NMN administration in various mouse models of human disease, several clinical trials administering NMN have been conducted recently [27]. Reports indicate that a single oral administration of NMN up to 500 mg was safe and effectively metabolized in healthy subjects without causing severe adverse events [48,49]. More interestingly, a 10 week, randomized, placebo-controlled, double-blind trial to evaluate the effect of NMN supplementation in postmenopausal women with prediabetes has shown NMN increases muscle insulin sensitivity and insulin signaling in prediabetic women [50].

## 5. Conclusions

We report that supplemented NMN can increase NAD^+^ in the mouse brain. Despite the tremendous research efforts aimed at exploiting the therapeutic potential of NMN to treat metabolic and aging-related diseases using dietary supplements, further research is suggested with regard to the prospects of developing drugs based on NMN [12]. In addition, human clinical trials are needed to explore the functional benefits of an increase in NAD^+^.

## Figures and Tables

**Table 1:** Effects of NMN administration and level of NAD^+^ in mice brain.

Mice Model	Intervention	Percentage of NAD^+^ Increased in Brain Tissues	Effects	Reference
Triple transgenic Alzheimer’s disease model mice	NMM (40 μg/g/day) for eight months	Unspecified	Reduced beta amyloid (Aβ), improved brain bioenergetics and preserved mitochondrial functionality.	Liu, et al. [36]
C57BL/6N	NMN (i.p. 500 mg/kg/day) single dose.	Hippocampal tissue; 34–39% within 15 min.	Unspecified	Stein and Imai [23]
C57BL/6N	NMN (drinking water; 100 or 300 mg/kg/day) for 12 months	Unspecified	Maintain neural stem/progenitor cells proliferation and self-renewal with age.	Stein and Imai [23]
PC12 cells (Parkinson’s disease cellular model)	NMN (0.1 mM to 1 mM). The treated cells were incubated for 24 h.	-	Reduced the rotenone-induced apoptosis and restored intracellular NAD^+^ level and ATP.	Lu, et al. [37]
C57BL/6N Adipose tissue-specific Nampt KO (ANKO)	NMN (i.p. 500 mg/kg /day) single dose	Individual hypothalamic nuclei (Arc, VMH, DMH, and LH); 1.5 to 3.5-fold increase 1 h after NMN administration.	Improved physical activity of the mice compared with control in the first half of the 12 h dark time.	Yoon, et al. [13]
APPswe/PS1dE9 (AD-Tg) mice	NMN (s.c. 100 mg/kg/day) for every other day for 28 days.	Forebrain tissue was examined after 24 h NMN injection; the % of increased NAD^+^ level was unspecified.	Increased mitochondrial respiratory function and decreased amyloid precursor protein (APP).	Long, et al. [38]
C57BL/6N	MNM (oral gavage; 300 mg/kg) single dose.	Cortex; ~10% increased within 60 min	Unspecified	Mills, et al. [15]
C57BL/6N	NMN (drinking water; 100 and 300 mg/kg/day) for 12 months.	Unspecified	Improved the rod cells functions in aged mice.	Mills, et al. [15]
C57BL/6	NMN (i.p. 62.5 mg/kg/day) Single dose.	Hippocampal tissue was examined; the % of increased NAD^+^ level was unspecified.	Ameliorated hippocampal CA1 injury.	Park, et al. [39]
Wister rat (Alzheimer’s diease model)	NMN (i.p. 500 mg/kg/day) for 10 days.	Hippocampal tissue was examined after the treatment; the % of increased NAD^+^ level was unspecified.	Restored the level of NAD^+^ and ATP; eliminated ROS accumulation in hippocampal tissue.	Wang, et al. [40]
APPswe/PS1dE9 double transgenic (AD-Tg) mice	NMN (s.c. 100 mg/kg/day) every other day for 28 days	Unspecified	Decreased β-amyloid production and increased cognitive function.	Yao, et al. [14]
C57BL/6 (CA1-specific Nampt knockdown mice)	NMN (oral gavage. 300 mg/kg/day) for three weeks.	Hippocampal tissue was examined; the % of increased NAD^+^ level was unspecified.	Increased level of NAD^+^ and improved cognitive function in old 20-month-old mice.	Johnson, et al. [41]
C57BL/6	NMN (i.p. 62.5 mg/kg/day) single dose.	Hippocampal tissue was examined after 24 h; the % of increased NAD^+^ level was unspecified.	Reduced mitochondrial fission and ROS in the hippocampus.	Klimova, et al. [42]
Wister rats	NMN (i.p. 100 mg/kg/day) every other day for 28 days.	Hippocampal and Prefrontal cortex tissue were examined; the % of increased NAD^+^ level was unspecified.	Alleviate aging-induced memory impairment; improved mitochondrial function and reduced apoptosis in brain tissues.	Hosseini, et al. [43]

i.p, intraperitoneal; s.c, subcutaneous; Arc, arcuate nucleus; VMH, ventromedial hypothalamus; DMH, dorsomedial hypothalamus; LH, lateral hypothalamus.

## Data Availability

Data supporting the reported results were generated during the study and are not publicly available. Summary of the results related to this study can be accommodated on request from the corresponding author.
